# Use of a Paid Digital Marketing Campaign to Promote a Mobile Health App to Encourage Parent-Engaged Developmental Monitoring: Implementation Study

**DOI:** 10.2196/34425

**Published:** 2022-04-05

**Authors:** Suraj Arshanapally, Katie Green, Karnesha Slaughter, Robert Muller, Demeika Wheaton

**Affiliations:** 1 Oak Ridge Institute of Science and Education Atlanta, GA United States; 2 National Center on Birth Defects and Developmental Disabilities Centers for Disease Control and Prevention Atlanta, GA United States; 3 Porter Novelli Atlanta, GA United States

**Keywords:** health communication, health promotion, internet, social media, child development, mobile health, pediatrics, parenting, early child development, developmental disability, mobile phone

## Abstract

**Background:**

The internet has become an increasingly popular medium for parents to obtain health information. More studies investigating the impact of paid digital marketing campaigns for parents on promoting children’s healthy development are needed.

**Objective:**

This study aims to explore the outcomes of a paid digital marketing campaign, which occurred from 2018 to 2020, to promote messages about parent-engaged developmental monitoring and ultimately direct parents to the Centers for Disease Control and Prevention’s (CDC’s) *Milestone Tracker* app, a mobile health (mHealth) app developed by the CDC.

**Methods:**

The paid digital marketing campaign occurred in 3 phases from 2018 to 2020. In each phase, 24 to 36 marketing messages, in English and Spanish, were created and disseminated using Google’s Universal App Campaigns and Facebook Ads Manager. Outcomes were measured using impressions, clicks, and install data. Return on investment was measured using click-through rate (CTR), cost per click, and cost per install metrics.

**Results:**

The Google-driven marketing messages garnered a total of 4,879,722 impressions (n=1,991,250, 40.81% for English and n=2,888,472, 59.19% for Spanish). The messages resulted in a total of 73,956 clicks (n=44,328, 59.94% for English and n=29,628, 40.06% for Spanish), with a total average CTR of 1.52% (2.22% for English and 1.03% for Spanish). From these clicks, there were 13,707 installs (n=9765, 71.24% for English and n=3942, 28.76% for Spanish) of the CDC’s *Milestone Tracker* app on Google Play Store. The total average cost per install was US $0.93 across all phases. The phase 3 headline “Track your child’s development” generated the highest CTR of 3.23% for both English and Spanish audiences. The Facebook-driven marketing messages garnered 2,434,320 impressions (n=1,612,934, 66.26% for English and n=821,386, 33.74% for Spanish). The messages resulted in 44,698 clicks (n=33,353, 74.62% for English and n=11,345, 25.38% for Spanish), with an average CTR of 1.84% (2.07% for English and 1.38% for Spanish). In all 3 phases, animated graphics generated the greatest number of clicks among both English and Spanish audiences on Facebook when compared with other types of images.

**Conclusions:**

These paid digital marketing campaigns can increase targeted message exposure about parent-engaged developmental monitoring and direct a parent audience to an mHealth app. Digital marketing platforms provide helpful metrics that can be used to assess the reach, engagement, and cost-effectiveness of this effort. The results from this study suggest that paid digital marketing can be an effective strategy and can inform future digital marketing activities to promote mHealth apps targeting parents of young children.

## Introduction

### Background

An estimated 1 in 6 children in the United States has a diagnosed developmental disability [1]. Developmental disabilities are conditions because of impairments in physical, learning, language, and behavioral domains. Early intervention of children with developmental disabilities can have a positive impact on their lives [2]. As a response, the Centers for Disease Control and Prevention (CDC) developed the *Learn the Signs. Act Early.* program, which aims to improve early identification of developmental delays and disabilities by facilitating parent-engaged developmental monitoring using developmental milestone checklists from birth through the age of 5 years. For many years, the CDC’s milestone checklists were offered as printed handouts and booklets. A Pew Research Center study [3] found that 85% of Americans owned a smartphone in 2021, which has increased from just 35% in 2011. The groups most dependent on smartphones include lower income Americans and those with a high school education or less. In addition, approximately half of parents with cell phones download apps on their mobile phones compared with one-third of nonparents [4]. Owing to the increasing smartphone use in America and the interest in mobile apps among parents, *Learn the Signs. Act Early.* developed the CDC’s *Milestone Tracker* mobile app, a mobile-friendly version of the milestone checklists. The app helps parents actively monitor their children’s developmental progress, sends notifications to parents about their child’s progress, and encourages parents to share any potential concerns with their child’s physician, a critical step toward early identification and connection with intervention services and supports.

Although traditional promotion using brochures and flyers have supported the promotion of the CDC’s *Milestone Tracker* app for a few years, there is mounting evidence that parents of young children learn about parenting and health information and resources through the internet and social media [5]. In a study conducted by Plantin and Daneback [6], first-time mothers aged 30 to 35 years were found to actively use the internet for health and parenting information and resources as well as social support. In addition, Facebook was reported as the most frequently used social media platform among parents seeking social support and health information on infant and child health [7].

The internet offers ample opportunities for promoting public health messages for many audiences, including parents of young children. There are easy-to-use digital marketing platforms such as Facebook and Google. Both platforms offer unpaid and paid placement options. However, the paid options increase the visibility of content, whereas the unpaid options do not, thereby increasing the likelihood of message exposure.

In 2014, Facebook announced that the mass production of content on their platform made it highly competitive for marketers. In their efforts to only serve the most valuable content to each user, most businesses and organizations saw a decline in their organic reach [8]. Ogilvy, a public relations firm, analyzed Facebook brand pages to assess the extent of the decline of organic (unpaid) reach [9]. Organic reach was defined as the number of people who saw the page’s post through their news feed or the page’s timeline or, in other words, anyone who saw content that was not a consequence of paid advertising. They selected 106 brand pages representing various industries, markets, countries, and sizes to obtain a comprehensive sample. The total audience reach for the sample was 48 million. In October 2013, the organic content from brand pages reached 12.05% of the total audience. By February 2014, there was a 49% drop, resulting in only 6.15% of the total audience being reached. In 2018, Facebook also announced that an update to their news feed algorithm would likely result in further decreases in organic reach [10]. This demonstrates the limits of organic reach, thus making the argument for methods such as paid digital marketing to increase the exposure to a message.

On Google, Yang and Ghose [11] found higher click-through rates (CTRs), which are the total clicks on a message divided by the total impressions, when paid content was available on Google search engines in addition to organic (unpaid) content, instead of organic content alone. As paid digital marketing plays an increasingly important role in reaching large audiences on the web, the need for more studies examining the effectiveness of such strategies in promoting health information and resources is warranted.

### Study Objective

This study assesses the outcomes of a paid digital marketing campaign that was conducted in 3 phases using both Google’s Universal App Campaigns (UAC) and Facebook Ads Manager to promote the CDC’s *Milestone Tracker* app among parents of young children.

## Methods

### Google’s UAC

Google’s UAC is a paid service to promote mobile apps. UAC distributes marketing messages across several Google formats and networks, such as the first page of relevant Google search results and small banner advertisements appearing on relevant YouTube channels. Google’s UAC also offers placement across the Google Display Network, which includes small banners next to videos on webpages and within apps in the Google Play Store, Gmail, and more [12]. This platform optimizes message performance by disseminating messages during certain times and in placements that the desired audience engages with the most.

### Facebook Ads Manager

Facebook Ads Manager is a paid advertising management service used to oversee paid digital marketing campaigns across the Facebook platform. Paid content is displayed within the Facebook news feeds as well as in between videos and news articles that appear when the user is scrolling [13]. The *Campaign Budget Optimization* feature within this service helps campaigns more efficiently spend the allocated budget by finding active opportunities that can help them achieve their desired results (eg, allocating more money toward top-performing messages and posting messages at times when audience engagement is high). Options to target parents who were already consuming and engaging with content related to young children were available and used as well.

### Campaign Design

The campaign occurred in 3 phases, each lasting 6 to 8 weeks. It began on June 6, 2018, and ended on June 14, 2020 (phase 1: June 6 to July 28, 2018; phase 2: March 27 to May 21, 2019; and phase 3: May 4 to June 14, 2020). The total campaign budget for the 3 phases, across both the Google and Facebook platforms, was US $24,500. The time frame and the allocated budget for each phase varied based on the availability of funding for each fiscal year. The first phase targeted only English-speaking audiences as only the English version of the CDC’s *Milestone* Tracker app was available at the time. In October 2018, the Spanish version of the app, *Sigamos el Desarrollo,* became available; thus, phases 2 and 3 targeted the promotion of the mobile health app to both English- and Spanish-speaking audiences. The paid digital marketing campaign on both Google and Facebook was managed by an Atlanta-based public relations firm, Porter Novelli (PN). After each phase, the CDC and PN reviewed the results and fielded the top-performing marketing messages in the next phase.

The CDC Plain Language framework [14] and CDC Health Equity Guiding Principles for Inclusive Communication framework [15] were used to ensure that the marketing messages were designed in a clear, accessible, and audience-specific manner. In total, 24 to 36 marketing messages were produced and implemented across each phase on each platform. Each message included 1 graphic (series of 2-3 rotating images [carousel], animated graphics interchange format [GIF], or static image) and 1 copy (text) combination. The graphics depicted young children from varying racial backgrounds aged <5 years, and the text included short, parent-friendly calls to action about tracking their children’s milestones and installing the CDC’s *Milestone Tracker* app.

**Figure 1:**
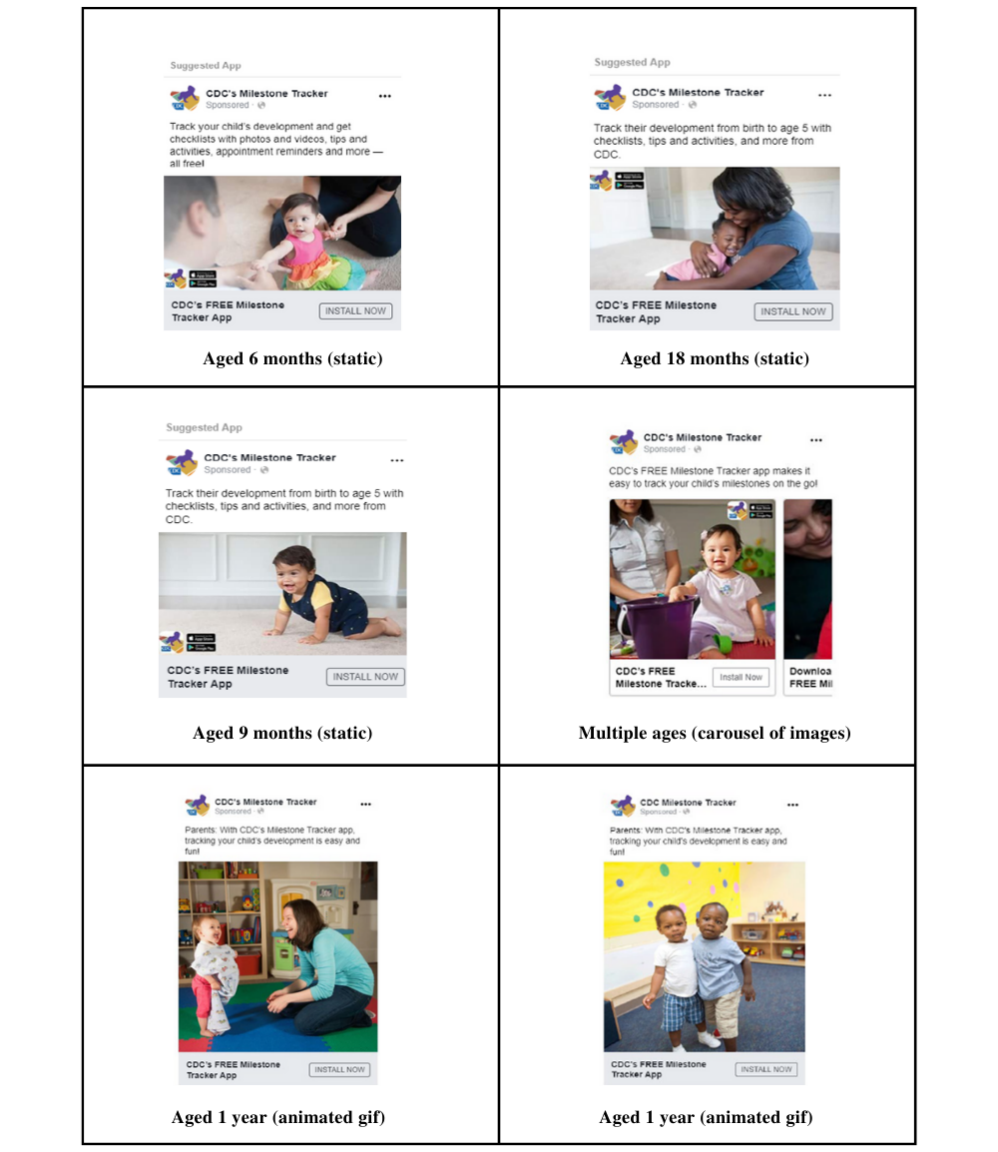
Screenshots of paid digital marketing posts on Facebook used in phases 1 to 3. GIF: graphics interchange format.

For Google, phase 1 included 24 English marketing messages, including 3 sets of copies. Both phases 2 and 3 included 36 English and Spanish marketing messages each, including 5 sets of copies. Either previews of the app or the app logo accompanied the copy. Owing to the platforms’ automated optimization features, only the highest performing marketing messages were used in each phase. For Facebook, phase 1 included 25 English marketing messages, including 5 sets of copies and 5 graphics (n=1, 20% carousel; n=1, 20% animated GIF; and n=3, 60% static images). Phase 2 included the same number of English marketing messages but also included 25 Spanish versions, including 5 sets of copies and 5 graphics (n=1, 20% carousel; n=2, 40% animated GIFs; and n=2, 40% static images). Phase 3 included 30 English marketing messages, including 5 sets of copies and 6 graphics (n=1, 17% carousel; n=2, 33% animated GIFs; and n=3, 50% static images), as well as 35 Spanish versions, including 5 sets of copies and 7 graphics (n=1, 14% carousel; n=1, 14% animated GIF; and n=5, 71% static images). All the marketing messages remained the same throughout the phases, except for 2 English graphics and 3 Spanish graphics from phase 2 to 3 and minor text updates. These updates were made to replace low-performing messages in the previous phases. All content was developed and reviewed by communications specialists at CDC and PN. Figure 1 includes just a sampling of the assets developed for this campaign. It demonstrates the racial and age diversity of the families and children depicted in the photos, as well as the various graphics used for optimization purposes. Each graphic was adapted for Spanish-speaking audiences as well.

### Audience Parameters

For Google-driven marketing messages, UAC uses machine learning to target users who are likely to install and use the CDC’s *Milestone Tracker* app: parents of young children. Google’s automated targeting strategy is based on user data collected by its platforms and properties, similar digital marketing campaigns, and current reach and engagement trends [10]. For instance, the marketing messages were served to users who searched keywords on children’s development.

In addition, Google and Apple do not share data between their respective platforms and properties; thus, Google-driven traffic to Apple’s App Store was not collected. Google-driven marketing messages were only served on Android devices using English and Android devices using Spanish, which is a limitation of the study.

On Facebook, the audience parameters for marketing messages were set for parents in the United States aged 18 to 45 years with young children aged <5 years. This included the following Facebook audience categories: parents of newborn babies, new parents (0-12 months), parents with toddlers (1-2 years), and parents with preschoolers (3-5 years). Separate sets were created for English- and Spanish-speaking parents. Marketing messages were distributed to four types of devices: Android devices using English, Android devices using Spanish, iOS devices using English, and iOS devices using Spanish. Therefore, when a user clicked on a marketing message, they were directed to either the Google Play Store or Apple’s App Store, depending on their device type.

### Data Collection

When users receive a Google- or Facebook-driven marketing message, they may click on the message. They will be directed to the App Store or Google Play Store, where they can install the CDC’s *Milestone Tracker* app. These steps are measured by the following metrics in Textbox 1.

**Definitions of digital marketing metrics.**

**Metric and definition**

- Impressions
  Number of times a paid digital marketing message is served to a user (includes repeat exposures)
- Click
  Action taken by a user upon seeing a paid digital marketing message to visit the App Store or Google Play Store to learn more about the Centers for Disease Control and Prevention’s (CDC’s) *Milestone Tracker* app
- Click-through rate
  Total number of clicks on the paid digital marketing message divided by the total number of impressions
- Cost per click
  Total cost of the paid digital marketing campaign divided by the number of clicks on a paid digital marketing message
- Install
  Downloads of the CDC’s *Milestone Tracker* app
- Install rate
  Total number of installations of the CDC’s *Milestone Tracker* app divided by the total number of clicks on the paid digital marketing message
- Cost per install
  Total cost of the paid digital marketing campaign divided by the number of installations of the CDC’s *Milestone Tracker* app

As Google shares information between platforms, including Google Display Network, YouTube, and Google Play Store, UAC could capture the number of Google-driven impressions, clicks, and installs on Android devices.

Facebook-driven marketing messages were served on both Apple and Android devices in both English and Spanish. However, install data were not available. To obtain install data from Facebook, the CDC’s *Milestone Tracker* app would need to be registered with Facebook, which includes the implementation of the Facebook Software Development Kit—a functionality that integrates Facebook into the app. As the CDC’s *Milestone Tracker* app must adhere to federal guidelines, this capability was restricted and thus served as a limitation in terms of data collection.

### Ethical Considerations

Institutional review board approval was not required for this project as no human participants were involved in the study, and the analyzed data were limited to publicly available digital metrics collected in aggregate.

## Results

### Impressions

Across all 3 phases, Google-driven marketing messages generated a total of 4,879,722 impressions, and Facebook-driven marketing messages generated 2,434,320 impressions. In phase 3, the CDC manually allocated more of the Facebook budget toward Spanish-speaking audiences as the *Campaign Budget Optimization* feature was allocating more funds toward and generating more impressions for English-speaking audiences in phase 2. For this reason, the number of Facebook-driven Spanish impressions was larger than English impressions in phase 3 (Table 1).

**Table 1:** Impressions from Google- (N=4,879,722) and Facebook-driven (N=2,434,320) marketing messages.

Campaign phases				Impressions, n (%)
**Phase 1 (2018)**				
	**Google^a^**			
		English	982,568 (20.14)	
	**Facebook**			
		English	626,382 (25.73)	
**Phase 2 (2019)**				
	**Google^a^**			
		English	676,628 (13.87)	
		Spanish	1,009,547 (20.68)	
		Total	1,686,175 (34.55)	
	**Facebook**			
		English	444,658 (18.27)	
		Spanish	217,465 (8.93)	
		Total	662,123 (27.2)	
**Phase 3 (2020)**				
	**Google^a^**			
		English	332,054 (6.8)	
		Spanish	1,878,925 (38.5)	
		Total	2,210,979 (45.31)	
	**Facebook**			
		English	541,894 (22.26)	
		Spanish	603,921 (24.81)	
		Total	1,145,815 (47.07)	

^a^For Google, the messages were only served on Android devices, not Apple devices, because of a tracking pixel needed to share data between Google and Apple.

### Clicks and CTR

During the campaign, the Google-driven marketing messages were clicked a total of 73,956 times (CTR 1.52%; Table 2). Although they were displayed across Google Search, Google Display Network, and YouTube properties, most of the impressions, clicks, and installs were driven by Google Display Network in all 3 phases (Table 3). In all 3 phases, the English messages had a higher CTR than the Spanish messages.

**Table 2:** Clicks, CTR^a,b^, and CPC^c,d^ metrics from Google- (N=73,956) and Facebook-driven (N=44,698) marketing messages.

Campaign phases				Clicks, n (%)		CTR (%)		CPC (US $)
**Phase 1 (2018)**								
	**Google^e^**							
		English	19,782 (26.75)		2.01		0.20	
	**Facebook**							
		English	11,822 (26.45)		1.89		0.30	
**Phase 2 (2019)**								
	**Google^e^**							
		English	16,284 (22.02)		2.41		0.18	
		Spanish	18,138 (24.53)		1.80		0.12	
		Total	34,422 (46.54)		2.04		0.15	
	**Facebook**							
		English	9711 (21.73)		2.18		0.34	
		Spanish	3595 (8.04)		1.65		0.34	
		Total	13,306 (29.77)		2.01		0.34	
**Phase 3 (2020)**								
	**Google^e^**							
		English	8262 (11.17)		2.49		0.23	
		Spanish	11,490 (15.54)		0.61		0.16	
		Total	19,752 (26.71)		0.89		0.19	
	**Facebook**							
		English	11,820 (26.44)		2.18		0.14	
		Spanish	7750 (17.34)		1.28		0.15	
		Total	19,750 (44.19)		1.71		0.19	

^a^CTR: click-through rate.

^b^Total number of clicks on the paid digital marketing message divided by the total number of impressions.

^c^CPC: cost per click.

^d^Total cost of the paid digital marketing campaign divided by the number of clicks on a marketing message.

^e^For Google, the messages were only served on Android devices, not Apple devices, because of a tracking pixel needed to share data between Google and Apple.

**Table 3:** Metrics^a^ for Google-driven marketing messages by Google platform placement.

Placements			Impressions (N=4,879,722), n (%)		Clicks (N=73,956), n (%)		CTR^b^ (%)		Installs (N=13,707), n (%)
**Phase 1 (2018)**									
	Google Search	20,236 (0.41)		1542 (2.09)		7.62		671 (4.9)	
	Google Display Network	962,257 (19.72)		18,226 (24.64)		1.89		2669 (19.47)	
	YouTube	75 (0)		14 (0.01)		18.67		0 (0)	
	Total	982,568 (20.14)		19,782 (26.75)		2.01		3340 (24.37)	
**Phase 2 (2019)**									
	Google Search	12,506 (0.26)		1027 (1.39)		8.21		442 (3.22)	
	Google Display Network	1,672,818 (34.28)		33,376 (45.13)		2.00		4218 (30.77)	
	YouTube	851 (0.02)		19 (0.03)		2.23		2 (0.01)	
	Total	1,686,175 (34.55)		34,422 (46.54)		2.04		4662 (34.01)	
**Phase 3 (2020)**									
	Google Search	18,828 (0.39)		1389 (1.88)		7.38		613 (4.47)	
	Google Display Network	2,191,913 (44.92)		18,357 (24.82)		0.84		5091 (37.14)	
	YouTube	238 (0)		6 (0.01)		2.52		1 (0.01)	
	Total	2,210,979 (45.31)		19,752 (26.71)		0.89		5705 (41.62)	

^a^Cost per click and cost per install metrics were not included. Google’s optimization features allocated varying amounts of the budget to each placement. Thus, individualized cost metrics could not be calculated.

^b^CTR: click-through rate.

Of the 73,956 clicks, during phase 1, the top-performing Google copy was “Track Milestones Today,” with 9288 (12.56%) clicks (CTR 2.97%); however, in phase 2, this headline was the second-best, with 3458 (4.68%) clicks (CTR 2.86%), whereas “Track Child Development” was the top-performing headline, with 4802 (6.49%) clicks (CTR 2.78%). In phase 3, the adapted phase 2 headline “Track your child’s development” generated the best CTR (3.23%).

The Facebook-driven marketing messages were clicked a total of 44,698 times (CTR 1.84%). In all 3 phases, the animated GIFs in both English and Spanish generated the greatest number of clicks when compared with other types of graphics, suggesting that the marketing messages formatted as animated GIFs may result in higher engagement than standalone static images. Of the 44,698 clicks, an image of 2 boys aged 1 year hugging from phase 3 received 8330 (18.64%) clicks (CTR 4.75%) singlehandedly, which made it the top-performing image among English marketing messages; images of babies aged 9 months resonated with both English and Spanish audiences, resulting in a total of 1797 (4.02%) clicks (CTR 0.91%) and 4175 (9.34%) clicks (CTR 1.19%) during phase 3, respectively.

### Cost per Click

For all 3 phases, the Google-driven marketing messages, on average, cost a total of US $0.17 per click (cost per click [CPC]). Specifically, it cost US $4000 to receive 19,782 clicks (CPC US $0.20) in phase 1, US $5000 to receive 34,422 clicks (CPC US $0.15) in phase 2, and US $3750 to receive 19,752 clicks (CPC US $0.19) in phase 3 (Tables 2 and 4).

Overall, the average Facebook-driven marketing messages cost a total of US $0.26 per click (CPC). They improved in efficiency from phase 2 to 3. In phase 2, it cost US $4500 to receive 13,306 clicks (CPC US $0.34), whereas in phase 3, it cost US $3750 to receive 19,572 clicks (CPC US $0.19). In phase 1, it cost US $3500 to receive 11,822 clicks (CPC US $0.30).

**Table 4:** Campaign budget breakdown.

Phases	Google (US $)	Facebook (US $)
Phase 1 (June 6 to July 28, 2018)	4000	3500
Phase 2 (March 27 to May 21, 2019)	5000	4500
Phase 3 (May 4 to June 14, 2020)	3750	3750

### Installs and Cost per Install

During the 7-week period of phase 1, there were 34,431 app installs, an 84% increase compared with 7 weeks before the paid digital marketing campaign began. After 7 weeks from the phase 1 end, there were only 19,391 app installs, a 44% decrease compared with when the paid digital marketing messages were running in phase 1 (Table 5). The Google-driven marketing messages contributed to 3340 app installs at a cost per install (CPI) of US $1.19 (Table 6). During the 8-week period of phase 2, there were 39,443 app installs, a 14% increase compared with 8 weeks before the paid digital marketing messages ran. After 8 weeks from phase 2, there were only 23,725 app installs, a 40% decrease compared with when the paid digital marketing messages were running in phase 2 (Table 5). The Google-driven marketing messages contributed to 4662 app installs at a CPI of US $1.07 (Table 6).

During the 6-week period of phase 3, there were 42,239 app installs. No major differences in app installs were found between phase 3 and the 6-week periods before and after the phase. The Google-driven marketing messages contributed to 5705 app installs at a CPI of US $0.66 (Table 6). For all 3 phases, the average CPI for Facebook could not be calculated because of the limitation of collecting Facebook-driven installs. The average CPI for Google across all 3 phases was US $0.93.

**Table 5:** Total CDC^a^ *Milestone Tracker* app install data.

Time point and period			Installs, n
**Before campaign**			
	7 weeks before	18,755	
	8 weeks before	34,730	
	6 weeks before	41,255	
**During campaign**			
	Phase 1^b^	34,431	
	Phase 2^c^	39,443	
	Phase 3^d^	42,239	
**After campaign**			
	7 weeks after	19,391	
	8 weeks after	23,725	
	6 weeks after	43,196	

^a^CDC: Centers for Disease Control and Prevention.

^b^Phase 1 occurred from June 6 to July 28, 2018, a 7-week period.

^c^Phase 2 occurred from March 27 to May 21, 2019, an 8-week period.

^d^Phase 3 occurred from May 4 to July 14, 2020, a 6-week period.

**Table 6:** Installs, install rate, and CPI^a,b^ metrics from Google- (N=13,707) and Facebook-driven marketing messages.

Campaign phases			Installs^c^, n (%)	Install rate (%)	CPI (US $)
**Phase 1 (2018)**					
	**Google^d^**				
		English	3340 (24.37)	16.89	1.19
	**Facebook**				
		English	—^d^	—	—
**Phase 2 (2019)**					
	**Google^d^**				
		English	3308 (24.13)	20.31	0.88
		Spanish	1354 (9.88)	7.46	1.62
		Total	4662 (34.01)	13.54	1.07
	**Facebook**				
		English	—	—	—
		Spanish	—	—	—
		Total	—	—	—
**Phase 3 (2020)**					
	**Google^e^**				
		English	3117 (22.74)	37.73	0.60
		Spanish	2588 (18.88)	22.52	0.73
		Total	5705 (41.62)	28.88	0.66
	**Facebook**				
		English	—	—	—
		Spanish	—	—	—
		Total	—	—	—

^a^CPI: cost per install.

^b^Total cost of the paid digital marketing campaign divided by the number of installs of the Centers for Disease Control and Prevention’s *Milestone Tracker* app.

^c^For Facebook, install data were not available because of federal guidelines against the integration of Facebook Software Development Kit into a mobile app.

^d^Data not available.

^e^For Google, the messages were only served on Android devices, not Apple devices, because of a tracking pixel needed to share data between Google and Apple.

## Discussion

### Principal Findings

This study investigated the outcomes of a paid digital marketing campaign for promoting Google- and Facebook-driven marketing messages about parent-engaged developmental monitoring and direct parents with children aged <5 years to the CDC’s *Milestone Tracker* app. Overall, the Google-driven marketing messages garnered a total of 4,879,722 impressions (n=1,991,250, 40.81% for English and n=2,888,472, 59.19% for Spanish). The messages resulted in a total of 73,956 clicks (n=44,328, 59.94% for English and n=29,628, 40.06% for Spanish), with a total average CTR of 1.52% (2.22% for English and 1.03% for Spanish). From these clicks, there were 13,707 installs (n=9765, 71.24% for English and n=3942, 28.76% for Spanish) of the CDC’s *Milestone Tracker* app on Google Play Store. The total average CPI was US $0.93 across all phases. The phase 3 headline, “Track your child’s development,” generated the highest CTR of 3.23% for both English and Spanish audiences. The Facebook-driven marketing messages garnered 2,434,320 impressions (n=1,612,934, 66.26% for English and n=821,386, 33.74% for Spanish). The messages resulted in 44,698 clicks (n=33,353, 74.62% for English and n=11,345, 25.38% for Spanish), with an average CTR of 1.84% (2.07% for English and 1.38% for Spanish). In all 3 phases, animated graphics generated the greatest number of clicks among both English and Spanish audiences on Facebook when compared with other types of images.

### Marketing Messages

Among the Facebook- and Google-driven marketing messages fielded through this campaign, the marketing messages with animated GIFs and images with younger children (eg, 2 boys aged 1 year hugging and a baby aged 9 months) performed higher than messages without GIFs and images of older children. The marketing messages with simple and direct calls to action, such as the “Track your child’s development” copy, generated high CTRs. Future public health campaigns targeting parents of young children can consider these findings when designing marketing messages for Facebook and Google.

### Return on Investment

The return on investment was measured by the increase in app installs during the paid digital marketing campaign compared with the periods before and after the campaign, when no paid digital marketing efforts were active. As no benchmarks for CPI for similar populations or studies were found for comparison, CTR and CPC were also analyzed. CTR provides additional information on the returns of the study, whereas CPC provides additional information on the cost-effectiveness of the investment. According to Table 5, the app installs during phases 1 and 2 were higher than the periods both before (84% and 14% higher, respectively) and after the campaign (44% and 40% higher, respectively). This demonstrates the effectiveness that the paid digital marketing campaign had on increasing app installs. The app installs during phase 3 remained consistent with the periods before and after the campaign, likely because of the competing health information on the web during the COVID-19 pandemic.

In total, the campaign generated 13,707 Google-driven installs, which excludes the app installs from Facebook. With a US $12,750 Google budget, the Google-driven marketing messages resulted in 13,707 users installing the CDC’s *Milestone Tracker* app.

The CTR depicted the effectiveness of a marketing message, particularly whether it could persuade the parents to click on the impression to learn more. The CPC depicted how cost-effective the campaign was in terms of clicks, particularly whether the campaign could receive a high number of clicks for a low cost. According to a study on industry-specific Google benchmarks, the benchmarks for the health and medical field were a CTR of 3.27% and CPC of US $2.62 for Google Search, as well as a CTR of 0.59% and a CPC of US $0.63 for the Google Display Network [16]. In a similar study on industry-specific Facebook benchmarks, the health care field showed a CTR of 0.83% and a CPC of US $1.32 [17]. For CTR, all Google-driven marketing messages, including Google Search and Google Display, outperformed industry standards (Table 3). Although CPC could not be calculated for Google Search and Google Display Network individually, the Google- and Facebook-driven marketing messages, in total, had significantly lower CPCs than those of industry standards (Table 2).

However, it is important to note that comparing CTR and CPC with industry-specific benchmarks for the whole health and medical field should not be the only method of evaluating effectiveness, as those benchmarks may be too broad. In addition, the focus of our campaign was not to sell a medical product but rather to promote public health messages to parents with young children. The *Campaign Budget Optimization* feature’s tendency to increase the efficiency of budget spending by disregarding lower performing devices and increasing delivery of impressions to higher performing devices is not ideal for public health purposes. It is crucial that messages about the CDC’s *Milestone Tracker* app are equitably promoted to all populations, which includes all device types regardless of performance. Thus, comparing CTR and CPC with marketing campaigns with similar populations, public health topics, and campaign goals may provide a more useful reference point than solely industry benchmarks.

For instance, Graham et al [18] conducted a similarly structured 3-phased paid digital marketing campaign using both Google- and Facebook-driven messages on healthy weight gain with expecting parents and parents of young children up to the age of 6 years in Alberta, Canada. Although their study differed in audience size, public health topic, and budget spending, their CTR and CPC could be used as reference points for evaluating the effectiveness of our campaign in terms of reaching similar audience demographics. On average, their Facebook-driven messages resulted in a CTR of 1.88% and a CPC of CAD $0.35 (US $0.26). Their Google-driven messages resulted in a CTR of 5.8% and CPC of CAD $0.76 (US $0.56). In comparison, our total average CTR for Facebook was 1.84%, and CPC was US $0.26, whereas for Google, the CTR was 1.52%, and CPC was US $0.17. A contributing reason for our Google CTR (1.52%) being much lower than their Google CTR (5.8%) was our study’s efforts to message Spanish-speaking audiences, a group that had lower Google CTRs (phase 2: 1.80%; phase 3: 1.65%) than the Google CTRs for English-speaking audiences (phase 2: 2.41%; phase 3: 2.49%; Table 2). Nevertheless, this paid digital marketing campaign was shown to be on par with industry benchmarks as well as a campaign with a similar public health approach. In addition, the metrics from this study can serve as a reference point for future digital marketing campaigns that target parents with children aged <5 years around topics related to parent-engaged developmental monitoring.

### Other Influences

In addition, it is important to note that the time frame for phase 3 overlapped with the beginning of the COVID-19 pandemic during a time when many consumers were searching for health-related apps but were also inundated with competing health information on the web.

### Limitations

There were multiple limitations to this study. First, only aggregate data were collected for this study. Therefore, it is not possible to know information about the users who installed the app. It cannot be confirmed that the app installs came from parents of young children. However, as Facebook- and Google-driven marketing messages only targeted parents of young children, they are the most likely audience to have installed the app because of the campaign. Second, the number of installs because of the Facebook-driven marketing messages could not be tracked. Thus, alternative measures, including CTR and CPC, were used as proxies. Third, mobile app use could not be tracked through this study.

### Conclusions

This study contributes to the literature by reporting on the outcomes and providing a cost analysis of using paid digital marketing campaigns on Google and Facebook to promote public health messages to parents of young children. As the internet and social media have become an increasingly popular medium of accessing health information, public health organizations should consider paid digital marketing as a tactic for reaching target audiences on the web.

## Abbreviations

CDC: Centers for Disease Control and Prevention
CPC: cost per click
CPI: cost per install
CTR: click-through rate
GIF: graphics interchange format
PN: Porter Novelli
UAC: Universal App Campaigns
